# Development of a targeted oral pharmacologic duodenal exclusion therapy for the treatment of metabolic diseases

**DOI:** 10.1126/sciadv.adu1326

**Published:** 2025-05-30

**Authors:** Taylor L. Carlson, Kevin Colbert, Marcela Vieira, Florence V. Guerina, Christine L. N. Bryant, Kirk Habegger, Pankaj Jay Pasricha, John Petersen, Steven Polomoscanik, Thomas H. Jozefiak, Ashish Nimgaonkar

**Affiliations:** ^1^ Glyscend Therapeutics Inc., Baltimore, MD, 21205, USA.; ^2^ Gastroenterology Imaging, Clario (eResearch Technology) , Philadelphia, PA 19103, USA.; ^3^ Department of Molecular Cell Biology and Biochemistry, Boston University, Boston, MA 02215, USA.; ^4^ Division of Endocrinology, University of Alabama at Birmingham, Birmingham, AL 35294, USA.; ^5^ Division of Gastroenterology, Department of Medicine, Mayo Clinic, Phoenix, AZ 85054, USA.; ^6^ Center for Bioengineering Innovation and Design, Department of Biomedical Engineering, The Johns Hopkins Hospital, Baltimore, MD 21287, USA.; ^7^ Division of Gastroenterology, Department of Medicine, The Johns Hopkins Hospital, Baltimore, MD 21287, USA.

## Abstract

Type 2 diabetes (T2D) and obesity are chronic metabolic diseases with global morbidity and mortality. Decades of evidence from surgical and endoscopic procedures bypassing the duodenum underscore the duodenum’s critical role in regulating glycemia and body weight. Although metabolic surgeries and endoscopic procedures are effective, their invasiveness, cost, and scalability limit patient access. We developed an orally administered mucin complexing polymeric (MCP) drug, designed to replicate duodenal exclusion physiology. MCPs, intended to have electrostatic and covalent cross-linkages with mucin glycoproteins, form extended network structures with resulting alteration of mucus barrier properties. Selective targeting of the duodenum is achieved via pH-based activation chemistry. Following screening for physiochemical properties, pharmacokinetics, and efficacy, GLY-200 emerged as the lead drug candidate replicating duodenal exclusion physiology with improved glycemia, reduced body weight, and modulation of gut hormones in rodent models. This targeted oral therapy holds promise for treatment of T2D and obesity by mimicking duodenal exclusion without the invasiveness of surgery or endoscopic procedures.

## Teaser

GLY-200, an oral drug designed to complex mucin, replicates duodenal exclusion physiology in animal models of T2D and obesity.

## INTRODUCTION

The proximal small intestine, specifically the duodenum, has emerged as a notable player in energy regulation and glucose homeostasis due to its nutrient-sensing, hormonal, and neuronal signaling mechanisms (*1*). Diet-induced alterations in duodenal epithelial morphology (e.g., villous hyperplasia, changes in enterocytes and enteroendocrine cell number, and intestinal cell mass) and subsequent functional adaptations are associated with the pathogenesis of metabolic disease (*2*). Patients with severe type 2 diabetes (T2D) and obesity exhibit duodenal dysfunction and morphologic changes, similar to ones described above, along with altered glucose absorption physiology leading to enhanced blood glucose, insulin, and glucagon and decreased glucagon-like peptide–1 (GLP-1) (*2*, *3*).

It is well established that metabolic surgeries have a profound impact on T2D, obesity, and related comorbidities. Surgical intervention can markedly improve glycemic control, promote substantial and durable weight loss, improve quality of life and cardiovascular outcomes, and reduce mortality. Roux-en-Y gastric bypass (RYGB) surgery remains the most effective therapeutic modality with reports of up to 68.3 and 86.1% of patients achieving durable weight loss and T2D remission, respectively, at 5 years (*4*–*7*). Given its profound results, metabolic surgery is included in the American Diabetes Association treatment algorithm for T2D and is recognized in the guidance as having a “superior” effect on glycemic control and cardiovascular risk reduction compared with nonsurgical interventions (*8*).

In RYGB, the stomach is made smaller and attached more distally to the jejunum. The bypassed portion of the stomach and duodenum are removed from the path of nutrient flow. This exclusion of the duodenum from contact with intraluminal chyme, known as duodenal exclusion, may, in part, contribute to the profound and sustained reduction in blood glucose and body weight (BW) observed with this procedure. Notably, immediate and major improvements in glycemic control are achieved by RYGB, well in advance of substantial weight loss (*9*–*11*). These effects are mediated via several mechanisms, including reduced nutrient intake and/or absorption, enhanced L cell secretion of gut peptides such as GLP-1 and peptide YY (PYY), changes in the levels and composition of bile acids, and potentially decreased secretion of unidentified duodenal factors that may promote insulin resistance and/or have detrimental effects on ß cell secretion (*9*–*11*). There are multiple mechanisms driving metabolic improvements after RYGB. While it remains undetermined which mechanisms are most important in various patient populations, bypassing some or most of the duodenum is a dominant mechanism mediating the improved metabolic physiology after RYGB (*11*).

Endoscopic approaches such as the duodenal-jejunal bypass liner (DJBL) and duodenal mucosal resurfacing (DMR) technology, while not as effective as RYGB, most directly validate the clinical relevance of duodenal exclusion physiology as they lead to robust effects on glucose and BW without the confounding effects of the more invasive surgical procedures that also involve alterations to the stomach. DJBL treatment in patients with obesity and T2D resulted in a hemoglobin A1c (HbA1c) decrease of 1.3%, total weight loss of 18.9%, and excess weight loss of 36.9% with increases in GLP-1 and PYY (*12*). DMR treatment was also associated with HbA1c reductions of 1.4 to 2.5% with a greater degree of glycemic effect seen with the treatment of longer duodenal segments (*12*, *13*). Nevertheless, the costs, risks, and scalability of metabolic surgical procedures and devices restrict their use to only patients with the most severe T2D or obesity despite clear evidence that these procedures can reduce long-term risks of microvascular and macrovascular complications as well as all-cause mortality (*14*–*18*) and improve outcomes even in patients with lower body mass index (*19*–*21*). Thus, a less invasive, safer, and preferably oral duodenal exclusion therapy that could be used early in the diabetes and obesity treatment continuum could have impactful long-term benefits to patients.

To recapitulate the mechanism of duodenal exclusion with an oral approach, we developed a mucus complexing polymeric (MCP) drug that is inactive at gastric pH but crosslinks mucin in the intestinal mucus layer at duodenal pH (≥5.0). Following oral administration, a mucus-drug crosslinked complex would be formed through electrostatic and covalent interactions with mucin at intestinal pH (pH 5.0 to 7.0), leading to enhancement of the natural barrier function of mucus in the duodenum. This therapeutic approach has the potential to deliver the benefits of duodenal exclusion observed with metabolic surgery and DJBL devices in a scalable, safer, noninvasive, and more patient-friendly manner.

Here, we describe the development, characterization, and selection of GLY-200, a lead MCP drug candidate selected for the treatment of T2D and obesity via the duodenal exclusion mechanism. MCPs were initially characterized in in vitro and ex vivo models, and their properties were optimized. The lead MCP, GLY-200, was then evaluated for pharmacokinetics (PK) and efficacy in rodent and canine models. GLY-200 is currently being developed for the treatment of T2D and obesity. In a phase 1 clinical trial, GLY-200 treatment appeared to be safe and well-tolerated (*22*) and resulted in improvements in postprandial glucose, appetite, and gut hormones in healthy participants.

## RESULTS

### Designing, synthesizing, and screening MCP candidates

**Fig. 1.**
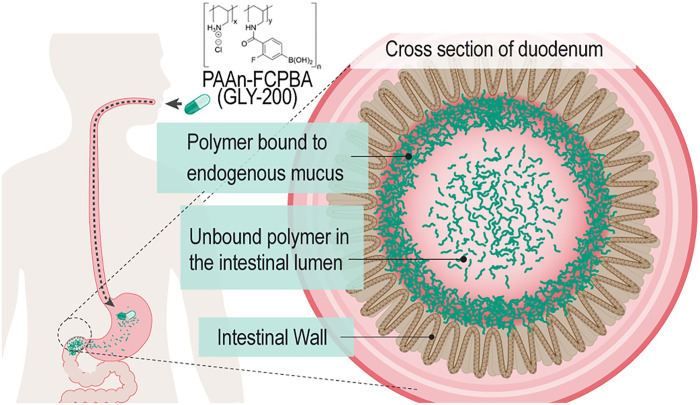
Schematic of oral polymeric drug capable of augmenting and enhancing the natural GI mucus barrier to emulate duodenal exclusion physiology.

Working from the understanding that mucus provides a barrier function in the gastrointestinal (GI) tract and that the physical properties of this barrier could be modulated by substances in the gut (*23*), we investigated mucin, a key component of mucus, as a pharmacological target for oral agents capable of augmenting and enhancing this natural barrier (Fig. 1). A focused discovery campaign to identify effective MCPs was guided by design criteria reflecting key drug attributes. These attributes include nonabsorbability from the GI tract, high solubility and stability in gastric and intestinal fluid, low viscosity to facilitate integration into the mucus layer, and formation of extended network structures through strong affinity with mucin glycoproteins, the main structural component of mucus.

In view of the design criteria outlined above, a discovery program was undertaken to evaluate polymers considered to have mucoadhesive or mucus-interacting properties. Mucin glycoprotein—with its high anionic charge, heavy glycosylation particularly with sialic acid, hydrophobic protein domains, and disulfide content—was considered as a molecular target. More than 75 polymeric compositions, naturally derived biopolymers and synthetic polymers were acquired and evaluated in functional assays for their ability to thicken or condense mucin solutions. Design criteria requiring both rapid dissolution and strong physical interactions with mucin solutions led this pursuit to cationic polymers, mostly those with high charge density. An optimization campaign followed which focused on the chemical modification of a set of six lead polycation backbones to produce multiple sublibraries of various sizes (6 to >100). Chemical modification approaches included alkylations, amidations, reductive aminations, Michael additions, and other reactions to alter the polymer’s solubility and ability to condense with mucin in solution. Free radical copolymerizations were also included for vinyl monomers of interest. This work led to a focus on polycations substituted with phenylboronic acid groups, known to interact strongly with sialic acids (*24*, *25*).

We found that poly(allylamine) (PAAn) could be modified with aryl carboxylic acid compounds, such as benzoic acid, 4-carboxyphenylboronic acid (CPBA), 3-fluoro-4-carboxyphenylboronic acid (FCPBA), and related compounds to produce modified polycationic compositions that retain high acidic and neutral solubility even at relatively high levels of substitution. These modified PAAn polymers were further characterized (Supplementary Methods) and shown to have high solubility (>200 mg/ml) and low viscosity [<20 cP at 10 weight % (wt %)] aiding quick integration into the mucus.

An initial screening step consisted of a mucin-mixing assay to identify polymers that were soluble and noninteractive in the gastric environment but complexed rapidly with mucin at intestinal pH. In this qualitative functional assay, a 1 wt % solution of porcine-derived mucin and a 1 wt % solution of test polymer were mixed in a vial or a well plate. Physical evidence of complexation, such as changes in optical clarity (clear, hazy, cloudy, and opaque) and physical state (solution, dispersion, suspension, precipitate, and phase-separated), were assessed. Compounds that reacted quickly (<1 min) and underwent a phase change at pH 5.5 were evaluated further.

To explore the physical properties of the polymer-mucin complexes, the mucin mixing assay was adapted to the filter cup of a centrifuge filter device. Centrifuge conditions were optimized to discriminate between polymer-mucin complexes based on their physical properties. Solutions or dispersions passed through the filter leaving very little residue in the cup (0 to 10% retained), while compositions exhibiting extended network barrier properties such as gels, precipitates, or films were retained in the cup along with substantial amounts of associated water. Compositions that resulted in ≥40% of the original solution mass being retained in the filter cup after centrifugation were considered promising.

**Fig. 2.**
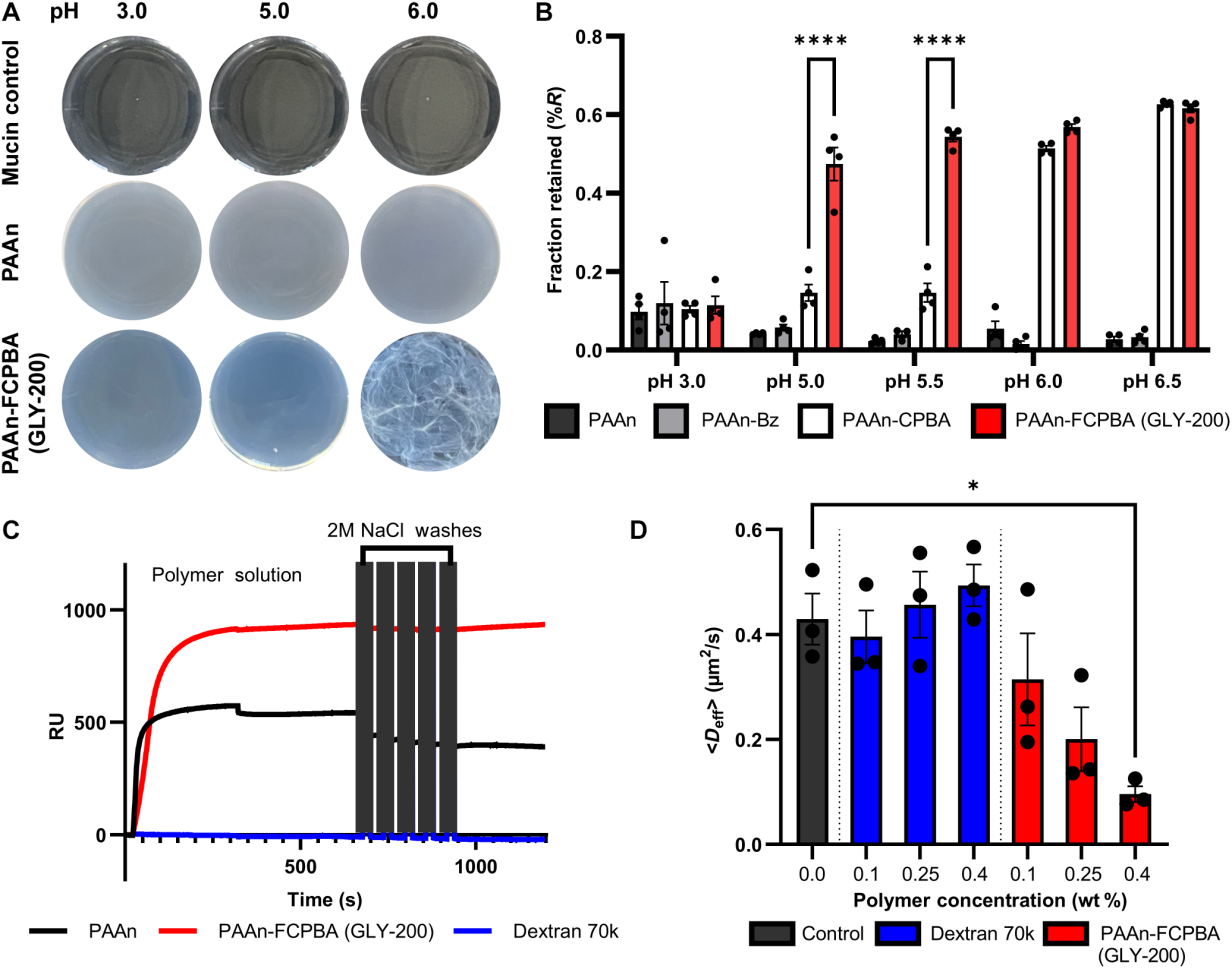
Targeted and pH sensitive mucus complexation. (**A**) Behavior of polymer with mucin solutions (1 wt %) was evaluated at different pH conditions to identify phase and appearance changes indicative of the formation of a polymer-mucin complex. (**B**) Mucin:polymer complex was quantified with a centrifuge assay to determine extended network barrier properties at different pH conditions (means ± SEM, *n* > 3). Significance is only shown between PAAn-CPBA and PAAn-FCPBA (****P ≤ 0.0001). (**C**) SPR sensogram identifies the binding behavior of polymer to mucin and evaluates the strength of the association following serial salt challenges (2 M NaCl) and (**D**) effective diffusivity (*D*_eff_) of particles in mucus treated with 0.0, 0.1, 0.25, and 0.4 wt % polymer. Trajectories of at least 300 particles were analyzed for each experiment group, and three separate experiments were performed to account for mucus variability. *P ≤ 0.05.

Poly(allylamine hydrochloride) modified with phenylboronic acid moieties such as CPBA or FCPBA produced polymers that were non-interactive at low pH values (pH 3 to 5) but rapidly complexed with mucin at intestinal pH conditions (pH 5 to 7). While unmodified PAAn formed a slightly cloudy dispersion when mixed with mucin at intestinal pH, PAAn modified with certain phenylboronic acids (e.g., PAAn-FCPBA) presented as opaque and sticky gels (Fig. 2A) with a large fraction (>40%) retained in the filter cup (Fig. 2B) at pH values of 5.0 to 6.0 depending on the modification. After these steps, PAAn-FCPBA, hereby referred to as GLY-200, was selected as the lead candidate based on its superior complexing activity in the duodenal pH range (pH 5 to 6).

GLY-200 is prepared via amide forming chemistry in a one-step synthetic modification of PAAn with excellent coupling efficiency (>99%) in aqueous solution using 1-ethyl-3-(3-dimethylaminopropyl)carbodiimide (EDC)/Hydroxybenzotriazole (HOBt) coupling reagents (fig. S1 and Supplementary Methods).

### Characterization of mucin-polymer complexes

Surface plasmon resonance (SPR) was used to evaluate the binding of PAAn and PAAn modified polymers on a mucin modified surface. Carboxy-functional SPR chips were covalently modified with mucin using a standard EDC/*N*-hydroxysuccinimide (NHS) chemistry protocol. The loading of mucin on the chip surface was controllable, and the mucin-modified surfaces were stable. Initial experiments demonstrated strong binding for PAAn and PAAn-modified polymers to the mucin-modified surfaces with minimal dissociation at pH 6, while Dextran 70k, a similarly sized multivalent analyte used as a negative control, did not associate with the mucin surface. This binding was consistent with strong polyvalent cationic interactions between the multivalent mucin ligand and the multivalent polymeric analyte.

The nature of these polymer-mucin interactions was further interrogated by flowing polymer over mucin-modified surfaces and then testing the resulting complex with high salt (2 M NaCl) conditions. Exposure to the high salt condition resulted in a ~26% decrease in mass of PAAn-mucin complexes, which was not observed in the case of GLY-200 (Fig. 2C). The resistance of GLY-200 to elution with ionic solvent suggests that there is covalent binding between the polymer and mucin, which does not occur with the unmodified PAAn backbone. The covalent binding results from the interaction of the boronic acids in GLY-200 with the diols of mucins’ monosaccharide sugars (e.g., sialic acid).

To evaluate the impact of GLY-200 on the mucus microenvironment, real-time multiple particle tracking (rtMPT), a microscopy technique that probes bulk transport and rheological properties in complex environments, was used. The addition of GLY-200 to porcine mucus resulted in a concentration-dependent decrease in microsphere mobility, indicating a more occlusive mucus barrier. Microsphere diffusion became constrained and immobilized as the concentration of GLY-200 was increased and the distribution of particle diffusion coefficients became bimodal, illustrating a progression from a high mobility environment to a low mobility environment. In contrast, microsphere diffusion in the presence of a dextran control (a concentration/viscosity match) was nearly unchanged, with slightly increased diffusivity as a function of concentration (Fig. 2D and fig. S2).

### Visualization of mucus complexation on intestinal tissues

**Fig. 3.**
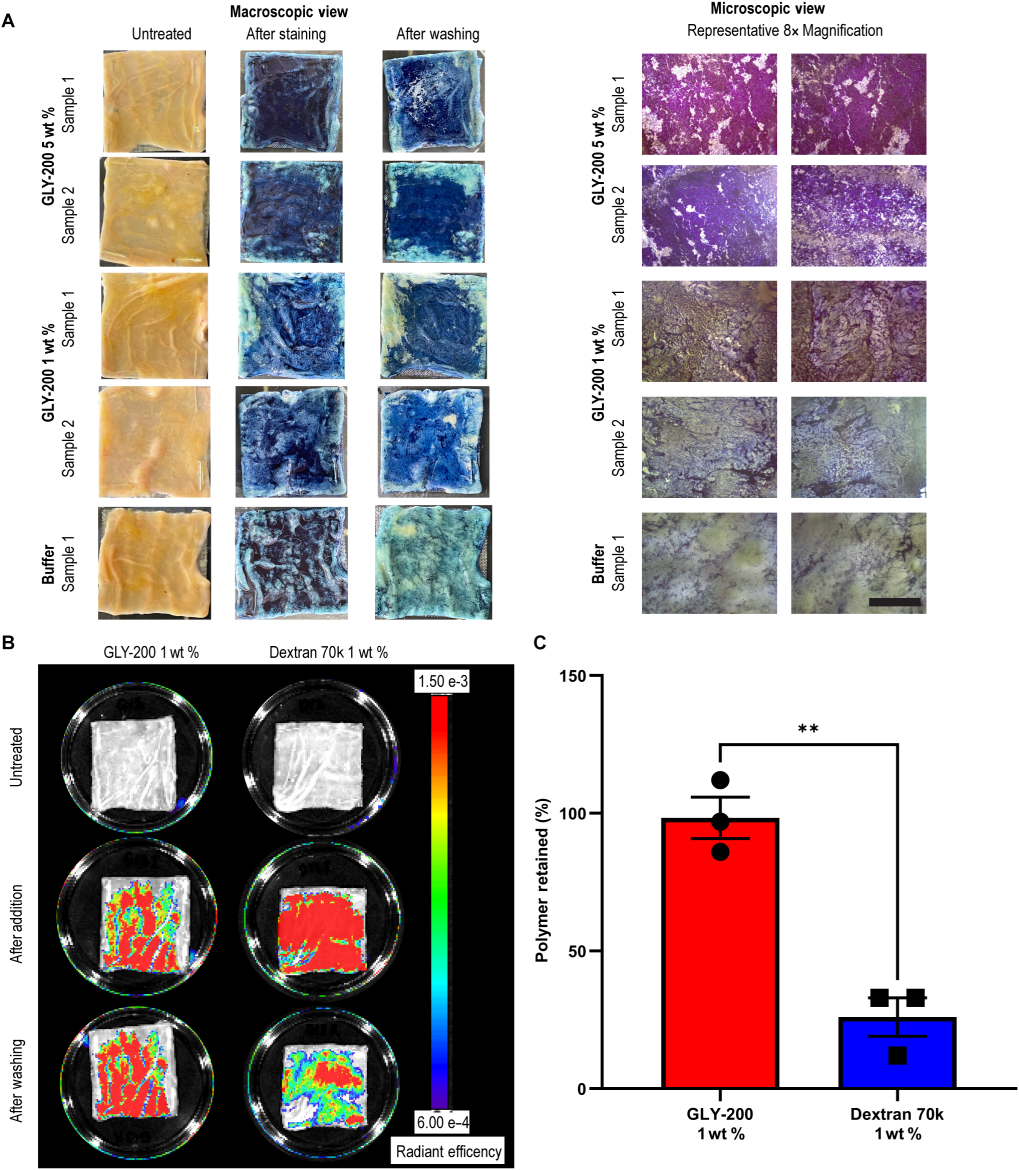
Robust, dose-dependent complexation on ex vivo intestinal tissue. (**A**) Macroscopic (5 cm by 5 cm) and microscopic images (8x magnification) of porcine tissue treated with GLY-200 or buffer visualize the GLY-200:mucus complex with an anionic dye, indigo carmine (complex retains indigo staining). Tissue was imaged before polymer or buffer addition, after incubation with dye, and after robust washing (*n* ≥ 4). Right: Two representative microscopic images (8x magnification) show tissues after washing. Scale bar, 0.5 cm. (**B**) IVIS images of porcine tissue (5 cm by 5 cm) treated with FITC–GLY-200 or FITC–Dextran 70k were taken before addition, after addition, and after robust washing, and (**C**) radiant efficiency from the IVIS images was measured and % retention is reported (*n* = 3).

GLY-200 complexation, coverage, and retention on intestinal tissue were tested ex vivo using porcine intestinal tissue. Tissue was treated with buffer (pH 6) or GLY-200, and complexed GLY-200 was visualized via staining using an anionic dye indigo carmine. The tissues then underwent a robust rinse cycle to remove unbound dye and measure retention of GLY-200. GLY-200 adhered to the intestinal mucus and was resistant to rinsing while the dye rinsed off buffer-treated tissue (Fig. 3A). GLY-200 evenly coated the treated intestinal surface. Higher magnification images show that the coverage of GLY-200 increases with higher concentrations, with a 5% GLY-200 solution displaying more consistent and darker staining than a 1% solution. In contrast, the buffer control did not reveal any distinct layer formation or network structure at higher magnification (Fig. 3A).

To further characterize the retention of the polymer-mucin complex, fluorescently labeled GLY-200 was evaluated using an In-Vivo Imaging System (IVIS). Quantitation of IVIS images shows GLY-200 complexed to mucin is highly retained (>80% retention) (Fig. 3, B and C) despite robust rinsing. In contrast, only 30% of Dextran 70k, a neutral (noncomplexing) polymer control, was retained after washing (Fig. 3, B and C). Similar results are observed with rat intestinal sections (fig. S3).

### Complexation and retention of GLY-200 in rat intestinal tract

To confirm that GLY-200 complexes with intestinal mucin when delivered orally and to better understand the transit of GLY-200 through the intestinal tract, rats were orally administered fluorescently labeled GLY-200 or Dextran 70k. IVIS and fluorescent immunohistochemistry (IHC) were used to compare the distribution and localization of GLY-200 or Dextran 70k in the GI tract at various time points after dosing.

**Fig. 4.**
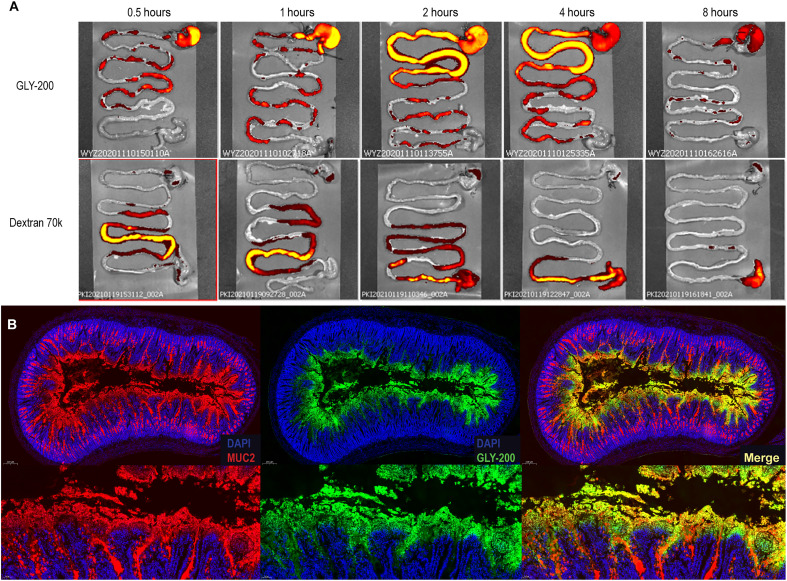
Intestinal transit and distribution of GLY-200. (**A**) Fasted rats were orally administered GLY-200 or Dextran 70k (80 mg per rat), and the GI tract was collected and imaged at 0.5, 1, 2, 4, and 8 hours to visualize polymer transit (*n* ≥ 2). (**B**) Sections of the proximal duodenum were collected and processed by IHC (4.7x and 17.3x magnification). The duodenal cross section shows colocalization of the polymer (green: GLY-200–FITC) and mucus (red: anti-MUC2;). DNA visualized with DAPI (blue).

Notably, IVIS results show that GLY-200 is present in the rat proximal small intestine for at least 4 hours, along with some retention in the stomach which may be due to delayed gastric and intestinal transit (Fig. 4A). This transit profile contrasts with Dextran 70k, which showed little to no presence in the duodenum at any of the profiled time points and had reached the ileum and exited the small intestine by 0.5 to 4 hours.

One limitation of IVIS is that the fluorescent signal is only captured in two dimensions, and it therefore does not distinguish between GLY-200 complexed with mucin on the intestinal mucus lining and GLY-200 (free or complexed with mucin) in the lumen. Therefore, to further interrogate whether GLY-200 is associated with the intestinal mucus lining, a section of the duodenum was collected and processed for IHC analysis. As seen in Fig. 4B, GLY-200 is prominent in the duodenum, coating the villus surface and colocalized with MUC2. The coating of villus surfaces and lack of crypt penetration suggest a rapid complexation of polymer with mucin, limiting polymer penetration that is supported by the complexation rate seen in vitro. This staining pattern was seen even after cross sections from ex vivo experiments were subjected to sequential rinses, demonstrating the integration and complexation of polymer into the mucus layer (fig. S4). Although it is likely that a portion of the IVIS signal can be attributed to luminal GLY-200, these data suggest that GLY-200 is bound to the mucus layer of the proximal small intestine and is likely resistant to shear stress.

### Gut restriction and fecal excretion of GLY-200

**Fig. 5.**
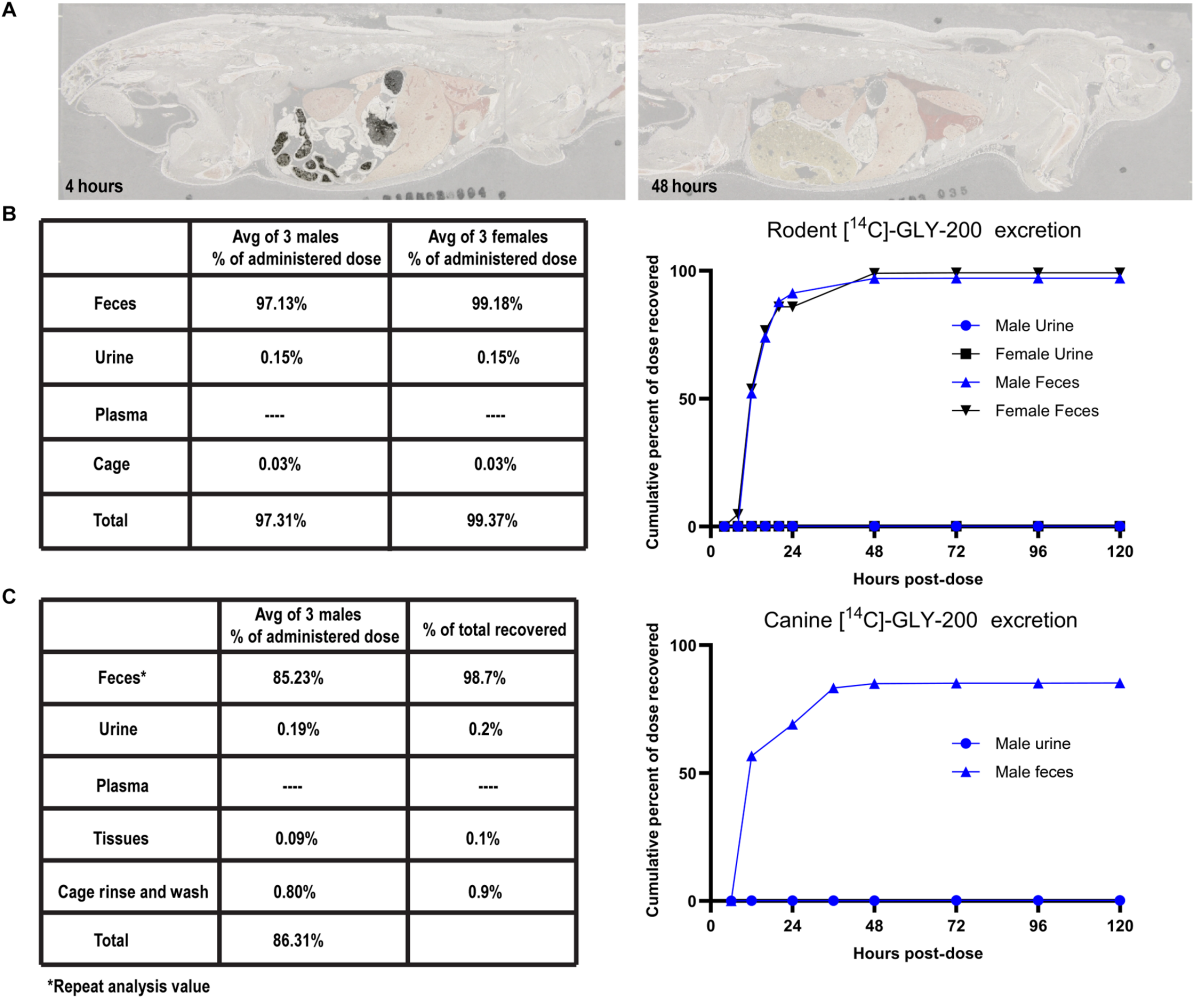
A PK, mass balance, and tissue distribution of GLY-200. (**A**) Whole body autoradioluminogram showing tissue distribution of radioactivity in rodent at 4 and 48 hours following an oral dose of [14C]-GLY-200 at 80 mg per animal (*n* = 1 per sex per time point). (**B** and **C**) Recovery of administered GLY-200 equivalents in (B) rodent and (C) canine following an oral dose of [14C]-GLY-200 (*n* ≥ 3).

GLY-200 is not expected to be systemically absorbed due to its molecular weight of approximately 60 kDa (polydispersity of 1.8), which exceeds the threshold for oral absorption. Moreover, the complexation of GLY-200 with mucus results in an insoluble complex that is excreted in the feces further reducing the likelihood of systemic absorption. To confirm, PK studies were conducted with GLY-200 in rodent and canine models. PK and excretion mass balance end points were measured using [14C]-radio-labeled absorption, distribution, metabolism, and excretion (ADME) with additional tissue distribution end points measured by quantitative whole-body autoradiography (QWBA) (Fig. 5A).

In rats, the total recovery of administered [14C]-GLY-200–derived radioactivity was 97.31% (males) to 99.37% (females). Nearly all the administered dose was excreted in feces. In male rats, 97.13% (feces) and 0.15% (urine) of the total administered [14C]-GLY-200–derived radioactivity was recovered over 120 hours post-dose (Fig. 5B). In female rats, 99.18% (feces) and 0.15% (urine) of the total administered [14C]-GLY-200–derived radioactivity was recovered more than 120 hours post-dose. Greater than 50% of the radioactivity in the excreta was recovered over 16 hours post-dose, greater than 85% of the radioactivity in the excreta was recovered over 20 hours post-dose, and greater than 95% of the radioactivity in the excreta was recovered over 48 hours post-dose. QWBA results showed containment of radioactivity within the GI tract.

In dogs, 85.23% of the total administered [14C]-GLY-200–derived radioactivity was excreted in feces which equates to 98.7% of the total recovered dose (Fig. 5C). Insignificant amounts were observed in the urine, cage rinse, or cage wash. Upon euthanasia, 0.09% of the dose remained in the GI tract. These data indicate that no appreciable amount of [14C]-GLY-200 was systemically absorbed, and the vast majority of the dose was present in feces. The less than complete target mass balance (>85% recovery) is expected in dog radiolabeled DMPK studies as it likely stems from challenges in fully collecting and homogenizing all fecal output (*26*).

### Reduction of postprandial hyperglycemia and BW gain in rat models of T2D and obesity

**Fig. 6.**
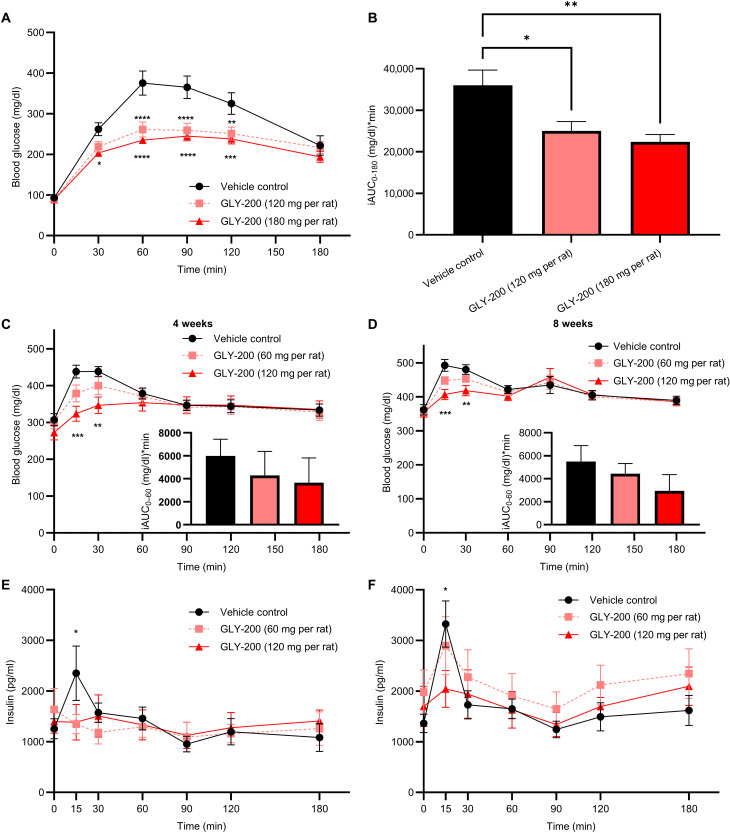
Postprandial glucose and insulin response following oral GLY-200 administration in rodent models. A standardized oral glucose tolerance test was performed on GK (**A** and **B**) and ZDF (**C** to **F**) rats. (A) Blood glucose and (B) iAUC_**0–180**_ response following a single dose of GLY-200 (120 and 180 mg per rat) in GK rats (*n* = 6 to 7). [(C) and (D)] Blood glucose and [(E) and (F)] insulin response in ZDF rodents receiving chronic GLY-200 treatment (60 and 120 mg per rat per day, *n* = 14 per group) for [(C) and (E)] 4 weeks and [(D) and (F)] 8 weeks. Means ± SEM, ****P ≤ 0.0001, ***P ≤ 0.001, **P ≤ 0.01, and *P ≤ 0.05.

The effect of a single dose of GLY-200 on glucose homeostasis was assessed in the Goto-Kakizaki (GK) rat model using a standard oral glucose tolerance test (OGTT). Male rats were treated with GLY-200 at two doses [120 mg per rat (~270 mpk) and 180 mg per rat (~400 mpk)] or 0.9% saline (vehicle control). As GLY-200 is nonabsorbed and gut-restricted, dosing a fixed amount as opposed to dosing by bodyweight was deemed appropriate as intestinal length is not meaningfully influenced by bodyweight (*27*). The administration of GLY-200 following an overnight fast resulted in a significant reduction in postprandial blood glucose at 60, 90, and 120 min post-meal (Fig. 6A). GLY-200 at 120 and 180 mg per rat produced significant reductions in the 180 minute incremental glucose area under the curve (iAUC_0–180_) of 31 and 38%, respectively, compared to vehicle (Fig. 6B).

The chronic metabolic effects of GLY-200 were then evaluated over 9 weeks of once-daily dosing in Zucker Diabetic Fatty (ZDF) rats (BW ~350 g, *n* = 14 per group). As there was no incremental efficacy benefit at the 180 mg per rat per day dose in the acute screening GK experiments, the doses of GLY-200 chosen for the chronic ZDF experiment were 60 mg per rat per day (~170 mpk), and 120 mg per rat per day (~340 mpk) compared to vehicle control. There were no significant changes in food intake or BW (fig. S5, A and B). Rats were dosed at the end of the light cycle following a short fasting period (3 to 4 hours). At week 4 and week 8, an OGTT was performed, and glucose and insulin were measured. The 120 mg per rat per day dose of GLY-200 significantly reduced postprandial glucose and insulin at week 4 and week 8 (Fig. 6, C to F). For the 60 and 120 mg per rat per day doses, respectively, incremental glucose AUC_0–60 min_ was reduced 28 and 39% at 4 weeks and 19 and 46% at 8 weeks (Fig. 6, C and D).

**Fig. 7.**
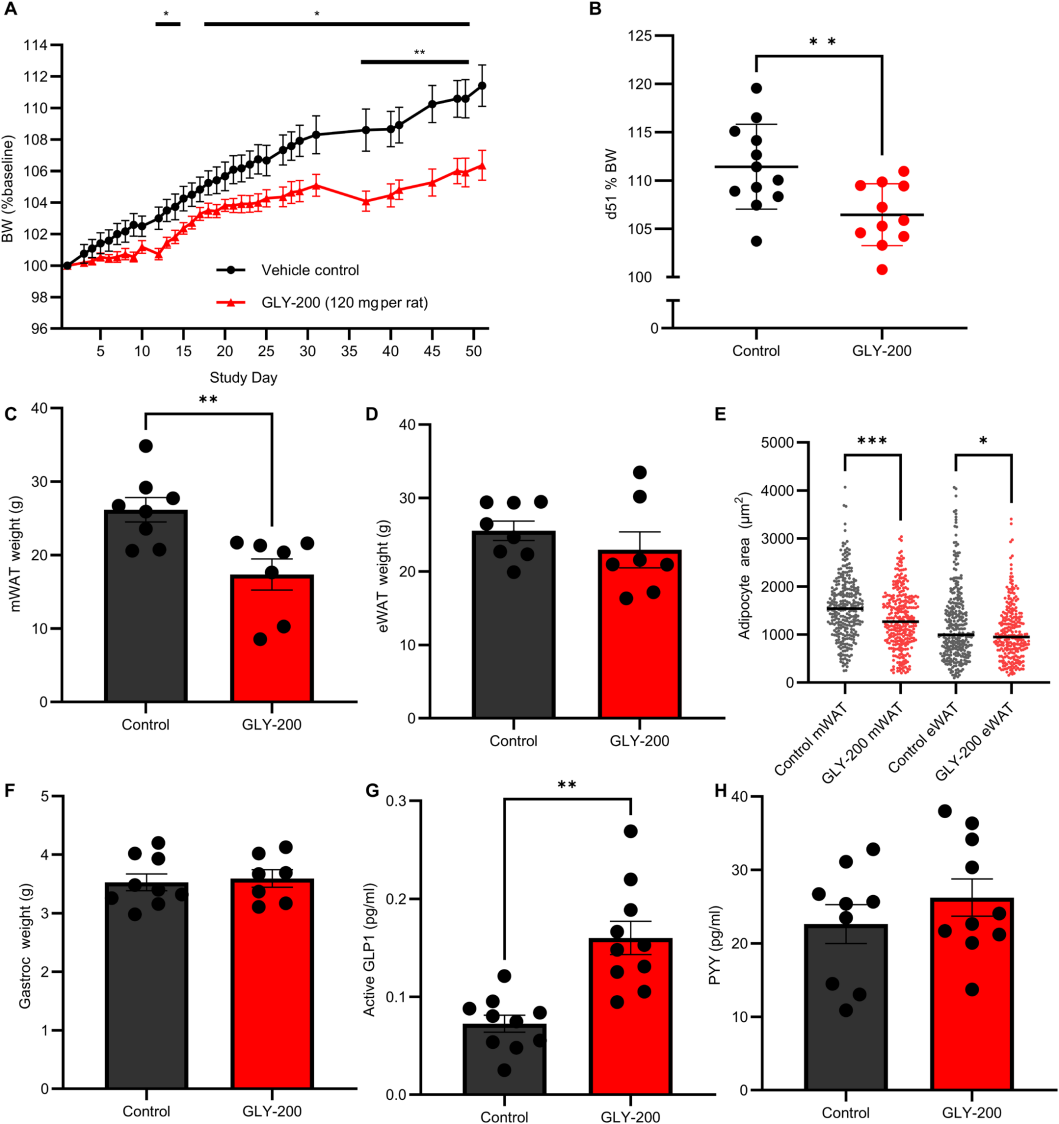
BW reduction, adiposity, and hormonal signals following once daily dosing of GLY-200 for 8 weeks in the DIO rat model. (**A**) GLY-200 resulted in significant reductions in BW gain by day 12 that further improved through day 51 (*P < 0.05 and **P < 0.01). *N* = 11 to 12 per group. Data are means ± SEM (A) and median (**B**), (**C**) mWAT, (**D**) eWAT, (**E**) adipocyte area, (**F**) gastric weight, (**G**) active GLP1, and (**H**) PYY evaluated in a subset of the animals (*n* ≥ 7). Data are means ± SEM. ***P ≤ 0.001, **P ≤ 0.01, and *P ≤ 0.05.

An 8-week diet-induced obesity (DIO) rat study evaluated the effect of once-daily administration of GLY-200 on BW. Obesity was induced in 24 male Sprague Dawley (SD) rats by feeding with high-fat diet (60% of caloric content from fat) for 8 weeks before the study start. Rats (BW ~800 g, *n* = 12 per group) were treated with either GLY-200 (120 mg per rat per day; ~150 mpk) or 0.9% saline vehicle control. Rats were dosed at the end of the light cycle following a short fasting period (3 to 4 hours). Treatment with GLY-200 resulted in significant reductions in BW gain by day 12 that was sustained through day 51 (last BW measurement), with a relative 6% lower weight gain compared to the control group (Fig. 7, A and B). There was no statistically significant difference in food consumption between GLY-200–treated and saline-treated animals (fig. S5C). Mesenteric white adipose tissue (mWAT) fat mass, an indicator of visceral adiposity, was significantly reduced in the GLY-200 treatment group compared to vehicle control (Fig. 7C). There was no statistically significant difference in the epididymal white adipose tissue (eWAT) fat mass or gastrocnemius muscle mass (Gastroc, a surrogate for lean muscle mass) between the treated and control group (Fig. 7, D to F). In mesenteric fat, adipocyte size was significantly reduced in GLY-200–treated rats compared to control rats (Fig. 7E). In addition, at the end of the study, there was a significant increase in fasting active GLP-1 (*P* < 0.01) and a trend toward increased PYY in animals treated with GLY-200 compared to vehicle control (Fig. 7, G and H).

## DISCUSSION

Diet-induced alterations in the duodenum, a vital organ in energy regulation and glucose homeostasis, and subsequent functional adaptations have been associated with systemic metabolic dysfunction (*2*). Surgical procedures (e.g., RYGB) and endoscopic technologies (e.g., DJBL) that bypass the duodenum (duodenal exclusion mechanism) induce immediate and sustained improvements in glycemic control along with durable weight loss (*28*). Surgical and endoscopic approaches to duodenal exclusion, although very effective, have limited patient access due to their invasiveness, cost, risks, and scalability.

To develop a noninvasive duodenal exclusion drug candidate, we took a unique approach at targeting the mucus layer of the duodenum. The mucus layer is a natural barrier that protects the GI epithelium from direct exposure to pathogens and endotoxins while allowing absorption of nutrients. It is a complex hydrogel composed of water (~90%), mucin, and assorted lipids, salts, DNA, and proteins. Mucins are the dominant structural component comprising approximately 3 to 5% of mucus. The physical properties of mucus (e.g., permeability, elasticity, and lubricity) are strongly influenced by mucin composition and glycosylation, which varies among the different mucus membranes of the body (i.e., eye, lung, vagina, and upper and lower GI tract). Mucin glycoproteins are characterized by a high content of sialic acids resulting in a net negative charge (*29*–*31*). Using the knowledge that the mucus barrier can be modulated and strengthened by exogenous agents (*23*), MCPs were specifically designed to integrate and cross-link mucus to form extended network structures to enhance its natural barrier properties with the goal of altering epithelial nutrient sensing and downstream metabolic regulatory physiology.

GLY-200 was selected as our lead compound as it exhibits many of the desired properties of an ideal MCP candidate. First, a compound must have rapid dissolution and dispersion in the stomach while having the ability to withstand the harsh gastric environment. GLY-200 has low viscosity (<15 cP at 10 wt %), high solubility (>200 mg/ml in water), and robust stability at low pH. GLY-200’s metabolic stability derives from the use of an aromatic carbonamide, which provides steric protection, making it an unlikely substrate for enzymes. Preliminary data-validated GLY-200 was stable to proteases and Cytochrome P450 (CYP) enzymes.

Second, upon entering the duodenum, a candidate must have the capability to form extended network structures with mucus and rapidly complex and integrate into the duodenal mucus layer. GLY-200 is uniquely designed to bind mucus as a cationic polymer modified with fluorophenylboronic acid, which targets the sialic acid sugars terminating mucin oligosaccharides through electrostatic and covalent interactions. The fluorophenylboronic acid functional group [p*K*_a_ (where *K*a is the acid dissociation constant) ~ 7.5] enables pH targeting of mucin above pH 5.0 (duodenal pH) and enables the rapid formation of condensed adherent films with extended network properties (Fig. 2). Upon transit into the duodenum, GLY-200 complexes with mucus on the intestinal wall and is resistant to shear forces within the GI tract. Ex vivo imaging confirmed the adherence and retention of GLY-200 on the duodenal mucus lining (Fig. 3). Duodenal targeting was further confirmed by in vivo imaging, which showed the retention of GLY-200 in the proximal small intestine for up to 4 hours longer than the transit of Dextran 70k, a negative control (Fig. 4). Thus, GLY-200 selectively targets the proximal small intestine as its site of action.

A third key feature of an MCP candidate is its nonabsorbability, which limits the potential for systemic off-target effects. GLY-200 achieves this via high–molecular weight (~60 kDa), integration into the mucus layer, and formation of an insoluble polymer-mucus complex. This complex is shed from the intestinal epithelial surface through natural mucus turnover and eliminated entirely through the feces. ADME studies in the rodent and canine demonstrate the absence of systemic absorption as substantially all the administered dose was found to be excreted in the feces, with no appreciable radioactivity in the plasma, urine, or tissues (Fig. 5).

To explore whether the observed complexation of GLY-200 with GI mucus had the intended physiological effect, the in vivo effects of GLY-200 were evaluated in three well-validated rodent models of metabolic disease: GK, ZDF, and DIO rats. Each of these models recapitulates a different aspect of the T2D and obesity pathophysiology. The GK model, a non-obese T2D rodent model, and the ZDF model, an obese T2D rodent model, are both used to evaluate hyperglycemia, hyperinsulinemia, and insulin resistance, while the DIO model is an obese rodent model with impaired glucose tolerance primarily used for assessment of BW changes. Treatment with GLY-200 was associated with improved glycemia in the GK and ZDF models. In GK rats, a single dose of GLY-200 was associated with a dose-dependent reduction of postprandial glucose (Fig. 6) and chronic glycemic efficacy was demonstrated in ZDF rodents at 4 and 8 weeks of treatment. GLY-200 blunts the postprandial glucose rise consistent with a potential acute effect on duodenal glucose absorption and/or delayed gastric emptying. In contrast, RYGB surgery is associated with accelerated gastric emptying due to the intended anatomical changes to the stomach leading to earlier *T*_max_. In the DIO rat model, GLY-200 treatment resulted in significant reductions in BW, mesenteric fat mass, and increased GLP-1 and PYY levels (Fig. 7). Unlike diet-induced SD rats, ZDF rats are hyperphagic and diabetogenic due to a defect in leptin signaling. It is plausible that the differences in BW response between the two models may be due to differences in the dominant metabolic pathways driving weight. The changes observed in these rodent models of metabolic disease are strongly supportive of duodenal exclusion physiology and are consistent with the findings from other studies, which investigated endoscopic DJBL intervention in rodent models (*13*, *32*, *33*).

There are translational limitations to this work as rodents and canines have known anatomic and physiological differences from humans in the GI tract including differences in pH, mucus thickness, and mucus clearance rate. In addition, given the complexities of rodent feeding habits (sleep-wake cycle, food eating pattern, etc.), the optimal dosing frequency and regimen could not be investigated in the rodent model. Dose frequency will be explored further in the clinic. Early clinical results in healthy adults show promise, with 5-day GLY-200 administration reducing postprandial glucose and insulin while increasing bile acids, GLP-1, PYY, and glicentin, consistent with duodenal exclusion physiology and indicating a pharmacological effect in the proximal small intestine (*13*, *22*).

In conclusion, we have developed a unique oral pharmacological approach to mimic the duodenal exclusion component of RYGB surgery and DJBL intervention. We describe our process for compound candidate selection and characterization in this report. Duodenal exclusion is a promising target for the treatment of T2D and obesity that may represent an orthogonal and potentially additive approach to existing therapies including GLP-1 receptor agonists. Validating this approach, other reports have described technologies aimed at coating or lining the surface of the intestinal lumen geared toward a similar therapeutic intent (*34*, *35*). However, to our knowledge, GLY-200 is the first oral pharmacologic duodenal exclusion drug with reported clinical proof of concept. Further development of GLY-200 for the treatment of diabetes and obesity is warranted.

## MATERIALS AND METHODS

Refer to Supplementary Materials and Methods for details regarding synthesis of mucin complexing polymers.

### Experimental design

The objective of this study was to identify a MCP with the ability to form an extended network with duodenal mucus and to emulate duodenal exclusion pharmacology. The ability of MCPs to complex mucus was evaluated at gastric and intestinal pH’s using functional assays to observe network formation (mucin mixing), quantify barrier properties (centrifuge assay), and determine the type of binding (SPR). MCP adhesion, retention, and durability on duodenal tissue were determined by staining the complex and by imaging the complex with an IVIS before and following a robust wash cycle. MCP transit, retention, and excretion were determined in vivo in rodents by IVIS imaging and in rodents and dogs by scintigraphy. Last, MCP efficacy was determined in three rodent models, GK, ZDF, and DIO by evaluating changes in blood glucose, BW, and gut hormones.

Animals were randomly assigned. Group and sample sizes for each experiment are indicated in the methods and figure legends. All animal studies and experimental procedures were approved by the Institutional Animal Care and Use Committee. Approvals are listed by the study type/animal model, ethics review committee, and study approval number: IVIS/rat, UMMS, PROTO202000118; ADME/rat, Charles River Laboratories, ADME/rat, 00718503 and ADME/dog, 00718504; Efficacy/ZDF rat, Gubra, GUS2020-030-GLY; efficacy/DIO rat, Johns Hopkins University, RA13M99; and efficacy/GK rat, Johns Hopkins University, RA13M99.

### Materials and reagents

Type-III porcine gastric mucin was obtained from Sigma-Aldrich (catalog no. 1778). Dextran (70 kDa, catalog no. D1449) and fluorescein-labeled dextran (70 kDa, catalog no. D1823) were obtained from Thermo Fisher Scientific. *N*,*N*′-dimethylethylenediamine (DMEN) was obtained from TCI America. Fluorescein isothiocyanate (FITC) was obtained from Acros Organics. All other materials were obtained from Sigma-Aldrich or Thermo Fisher Scientific unless noted otherwise.

### Synthesis of dye-labeled polymers

Polymers were labeled with fluorescein (FITC; ~2.5 mol % relative to whole monomer) for imaging studies. Briefly, the polymer was dissolved in Milli-Q water, and the pH was adjusted to 6.0. FITC was dissolved in anhydrous methanol (0.02 g/ml) before addition to the polymer solution. Following an overnight incubation in the dark, the solution was filtered with a 10-μm filter and exhaustively dialyzed (molecular weight cut-off: 6000 to 8000 regenerative cellulose membrane) in acidic water (pH 2.5). The acidic bag contents then underwent an aqueous-organic extraction with ethyl acetate to remove free fluorescein. The aqueous layer was isolated and lyophilized yielding an orange solid. The absence of significant quantities of free fluorescein was confirmed by high-performance liquid chromatography analysis.

### Purification of porcine gastric mucin

A water-soluble fraction of commercial porcine gastric mucin (Sigma-Aldrich, catalog no. 1778) was obtained by extraction of the commercial product. Briefly, mucin was dissolved in deionized water overnight, and the soluble fraction was separated by centrifugation at 5300 rpm for 60 min. The collected fraction was lyophilized and stored at −20°C until use.

### Mucin: Polymer complexation functional assay

Purified soluble mucin (1.0 wt %) and test polymer (1.0 wt %) in 0.1 M MES, 0.9% NaCl buffer (pH 5.5 to 6.5); 0.1 M sodium acetate, 0.9% NaCl buffer (pH 5.0); or 0.1 M glycine 0.9% NaCl buffer (pH 3.0) were mixed in a 1:1 volumetric ratio, and the change in appearance was recorded after 5 min (e.g., clarity and physical state).

### Centrifuge assay

Polymer: mucin complexation was quantified using a centrifuge assay. Test polymer and purified mucin solutions (1 wt% in buffer; pH 3.0, 5 to 6.0) were added to the filter cup of a pretared COSTAR, Spin-X Centrifuge Tube (filter: 0.45-μm cellulose acetate, Corning Inc.) in a 1:1 ratio. The tubes were incubated on a shaker at 37°C and 300 rpm for 30 min then centrifuged at 6000*g* for 12 min to separate the mucin-polymer complex. The filtrate weight was measured, and the fraction retained (%*R*) was calculated

%R=(Filtrate Weight−Starting Solution Weight)*100 (1)

### Surface plasmon resonance

All SPR analyses were performed on a BI-4500 5-channel SPR system from Biosensing Instruments (Tempe, Arizona). Zwitterionic SPR chips (ZC30M, Xantec, GmbH, Düsseldorf, Germany) were used in conjunction with a cationic blocking agent (DMEN) to reduce nonspecific interactions. The chips were first modified with type-III porcine gastric mucin using amine coupling chemistry with EDC/NHS reagents. Remaining reactive sites were blocked with DMEN, and the surface was precleaned with 20 mM NaOH. Reference channels containing only immobilized DMEN were also included to monitor non-specific binding and bulk refractive index changes. All subsequent binding experiments were performed in 10 mM Hepes-buffered saline (with 0.005% P20, pH 6).

Salt effects on polymer-mucin complex stability were evaluated by flowing a single concentration (7 μg/ml) of each polymer over mucin-modified surfaces. The complexes were then exposed to pulses of 2 M NaCl.

### Real-time multiple particle tracking

Transport through mucus was determined using multiple particle tracking (rtMPT). Briefly, porcine mucus was added to an eight-well chamber plate and neutral fluorescent nanoparticles (PEG-ylated Fluospheres, 200 nm, 0.0025% solids) in the presence of polymer (0.1 to 0.4 wt %) or 0.1 M MES (pH 6.0) buffer were added dropwise to mucus. After incubation (2 hours) in a humid dark chamber, particle movement was monitored by taking 20-s videos with a frame interval of 0.033 s. Videos were analyzed with MATLAB (*36*) to isolate particle trajectories and calculate mean square displacement (MSD; Eq. 2) and effective diffusivity (*D*_eff_; Eq. 3).

$$\text{MSD}={\left[x\left(t+\tau \right)-x\left(t\right)\right]}^{2}+{\left[y\left(t+\tau \right)-y\left(t\right)\right]}^{2}$$ (2)

$$D_{\text{eff}}=\text{MSD}/\text{4τ}$$ (3)

where *x*(*t*) and *y*(*t*) represent the particle coordinates at a given time and τ is the timescale. Microsphere diffusion measurements were performed using mucus from three different animals with >100 particles analyzed per group per mucus sample. Trajectories of at least 300 particles were analyzed for each experiment group, and three separate experiments were performed to account for mucus variability. Dextran was used as a concentration/viscosity match and negative control.

### Ex vivo mucus polymer complexation on porcine tissue

For polymer, mucus complexation on tissue was visualized by staining with indigo carmine. Briefly, porcine small intestine was obtained from a local abattoir within 2 hours of tissue collection (*n* = 2). The proximal intestine was cut into 5-cm lengths, opened, secured on a 5 cm–by–5 cm wire mesh, and rinsed with buffer [0.1 M MES 0.9% NaCl (pH 6)]. Polymer solutions (1 and 5 wt %) were added to the mucosal layer, and tissue was incubated for 10 min (*n* ≥ 3). The tissue was dipped in buffer to remove unbound polymer and then placed in indigo carmine solution for 10 min followed by three buffer rinse steps (2 min, 300 rpm). Samples were imaged before polymer addition, post-incubation in dye, and after rinsing to visualize polymer complexation and coverage. Higher magnification microscope images were taken with a Leica EZ4W microscope at 8x magnification. The assay was validated by confirming staining of the GLY-200:mucin complex in a simple system consisting of only mucin and polymer and the use of a buffer tissue control to confirm selectivity of dye to polymer over tissue.

### Mucus retention test of polymer on porcine and rat tissue

To evaluate polymer retention on tissue, rat and porcine tissue were treated with polymer followed by robust rinse steps. IVIS imaging was used to capture and quantify polymer by imaging tissue before polymer addition, after adding, and after rinsing. Polymer retention was calculated from measuring the total radiant efficiency of the polymer. %Polymer Retention = (after rinsing-original)/(after adding-original) × 100. Briefly, porcine small intestine (proximal 2 ft) was obtained from a local abattoir within 2 hours of tissue collection (*n* = 2). The proximal intestine was cut into 5-cm lengths, opened longitudinally, secured on a 5 cm–by–5 cm wire mesh, and rinsed with buffer (0.1 M MES 0.9% NaCl (pH 6). Polymer solutions (1 wt %) were added to the mucosal layer, and tissue was incubated for 10 min. The tissue was rinsed in buffer for 10 min at 300 rpm. At least three sections of tissue were used to test each condition. Rat tissue was obtained from overnight fasted rats immediately following euthanasia. The first half of the small intestine was divided into 8-cm lengths. Each section was filled with polymer, incubated 10 min, and then rinsed with buffer. At least three sections of tissue were used to test each condition. Following imaging, a section of the intestine (1 cm) was placed in Carnoy’s Fixative (60% ethanol, 30% chloroform, and 10% glacial acetic acid). Fixed samples were embedded in paraffin, sectioned, and immuno-stained for Muc2 (MUC2 GB11344) and Dapi by iHisto Inc. (Salem, MA). Slides were scanned with Panoramic MIKI II scanner (40×; 0.26 μm/pixel) using the FITC, 4′,6-diamidino-2-phenylindole (DAPI), and CY3 filter.

### IVIS in vivo imaging and histology

SD male rats (250 g; Charles River Laboratories) were fed a special low autofluorescence purified diet (Envigo, TD. 97184) for 7 days before the study start. The animals were fasted 12 hours before and during the study with free access to water. FITC-labeled polymers were administered by oral gavage (80 mg per rat in 1 ml of saline). The animals (*n* ≥ 2) were euthanized at sequential time points (0.5, 1, 2, 4, and 8 hours), and the intestine (stomach to cecum) was removed and imaged with a PerkinElmer IVIS. Following imaging, a section of the duodenum (1 cm) was placed in Carnoy’s fixative. Fixed samples were embedded in paraffin, sectioned, and immuno-stained for Muc2 (MUC2 GB11344) and DAPI by iHisto Inc. (Salem, MA). Slides were scanned with Panoramic MIKI II scanner (40×; 0.26 μm per pixel) using the FITC, DAPI, and CY3 filter.

### PK, mass balance, and tissue distribution studies

For the plasma PK phase, four male and four female Crl:CD(SD, 250 g, Charles River Laboratories) rats received five daily oral doses of GLY-200 at 80 mg per animal followed by a single oral dose of [14C]-GLY-200 at 80 mg per animal and a target radioactivity of 25 μCi per animal. Following radio-labeled dosing, blood samples were collected from two animals per sex at select time points through 48 hours. For the excretion mass balance phase, three male and three female Crl:CD(SD) rats received five daily oral doses of GLY-200 at 80 mg per animal followed by a single oral dose of [14C]-GLY-200 at 80 mg per animal and a target radioactivity of 25 μCi per animal. Following radio-labeled dosing, animals were placed into metabolism cages for separate collection of urine and feces through 120 hours. For the tissue distribution phase, six male and six female Crl:CD(SD) rats received five daily oral doses of GLY-200 at 80 mg per animal followed by a single oral dose of [14C]-GLY-200 at 80 mg per animal and a target radioactivity of 25 μCi per animal. At select time points post-radiolabeled dose, one animal per sex was anesthetized by isoflurane inhalation, and a blood sample was collected. Following blood collection, animals were euthanized by CO_2_ inhalation and carcasses processed for QWBA. Plasma, urine, feces, cage rinse, and cage wash samples were analyzed for total radioactivity by liquid scintillation counting (LSC).

Three male Beagle dogs (9 M, Charles River Laboratories) received five daily oral doses of GLY-200 at 500 mg per animal, followed by a single oral dose of [14C]-GLY-200 at 500 mg per animal with a target radioactivity of 1000 μCi per animal. Blood samples were collected from all animals pre–radio-labeled dose (*t* = 0 hours) and at approximately *t* = 0.17, 0.25, 0.50, 1, 2, 4, 6, 24, 48, 72, 96, and 120 hours post–radio-labeled dos and processed to plasma. Urine and feces were collected from all animals overnight before radio-labeled dosing (*t* = 0 hours) and at approximately *t* = 6, 12, 24, 36, 48, 72, 96, and 120 hours post–radio-labeled dose. Select tissues were collected following euthanasia at ~120 hours post–radio-labeled dose. Plasma, urine, feces, tissues, cage rinse, and cage wash were analyzed for total radioactivity by LSC.

### Standardized OGTT protocol

A standardized OGTT protocol was used to evaluate GLY-200 efficacy. Before testing, all rats were fasted overnight for 12 to 15 hours with access to water. Rats were split into test groups: control (group A), which received 0.9% saline, and treatment (groups B, C, etc.), which received test polymers predissolved in saline at a designated concentration. All rats received a standard 1.5-ml volume of test solution delivered by oral gavage administered in an identical fashion. Baseline blood glucose levels were taken 60 min after administration of polymer or vehicle control. Then oral gavage of a 40% glucose solution (2.0 g/kg rat) was given immediately after recording the baseline, *t* = 0, blood glucose measurement. Blood samples were taken from each rat at *t* = 30, 60, 90, 120, and 180 min following glucose administration. Because of the hypermetabolic state, ZDF rats were semi-fasted for 21 hours and then fully fasted for 3 to 4 hours before the OGTT.

### Pharmacology rat models

#### GK rat model

GK rats (BW ~450 g) were randomized into three groups (*n* = 6 to 7 per group) and fasted overnight. Groups received a 1.5-ml volume of 0.9% saline (vehicle control), GLY-200 (120 mg per rat; ~270 mg/kg), or GLY-200 (180 mg per rat; ~400 mg/kg). Then, the standardized OGTT protocol was followed. Post-prandial glycemic effect was the only endpoint measured in this study.

#### ZDF rat model

Male ZDF rats (*n* = 56, 6 week; Charles River Laboratories) were fed Purina 5008 diet from age 6 weeks and throughout the study. Weekly of 4-hour fasting blood glucose (FBG) was measured and once >80% of the rats presented 4hFBG levels > 10 mM, animals were randomized into four treatment groups (*n* = 14) based on 4-hour FBG and BW (week −1). Baseline samples were obtained before study start and 9 weeks treatment (PO, once daily) with vehicle or GLY-200. During the 9-week daily dosing study period, all rats were fasted for 3 to 4 hours every day (a.m.) and received once daily dosing. Rats were treated with vehicle, GLY-200 (60 mg/day; ~170 mg/kg), or GLY-200 (120 mg/day; ~340 mg/kg). In week 4 and week 8, an OGTT with insulin curve was performed. BW and food consumption were recorded approximately daily.

#### DIO rat model

Male SD rats were fed a high fat diet (60% kcal fat; D12492, Research Diets) for a 10-week lead-in period before the start of the study and throughout the 8-week daily dosing period. During the 8-week daily dosing study period, all rats were fasted from 2 to 5 p.m. every day and received once daily dosing between 5 and 6 p.m. every day. Once daily dosing consisted of a 1.5-ml oral gavage of GLY-200 at 120 mg per rat per day (150 mg/kg) or 0.9% saline. BW and food consumption were recorded approximately daily for ~7 weeks. FBG was recorded weekly. At the end of the study, rats were euthanized under isoflurane anesthesia. Gastrocnemius, mesenteric, and epididymal adipose tissue was collected, weighed, and then fixed in formalin; processed for paraffin embedding; and sectioned (*n* ≥ 7). Samples were stained with hematoxylin and eosin and imaged at ×10 magnification. Adipose size was measured using ImageJ (~300 adipocytes were measured for each treatment; *n* = 3). Terminal plasma was collected, and GLP1 and PYY levels were measured (*n* ≥ 7). A single animal from the GLY-200 group was removed due to excess BW loss identified as an outlier.

### Statistical analysis

Statistical analysis was performed with the aid of GraphPad Prism (GraphPad Software).

Significance for the centrifuge assay was determined using two-way analysis of variance (ANOVA) with Turkey’s post hoc test for multiple groups. rtMPT significance was determined using one-way ANOVA with Turkey’s post hoc test for multiple groups. Standard unpaired *t* test was used to evaluate GLY-200 versus control retention. Unless noted all values are expressed as means ± SEM, ****P ≤ 0.0001, ***P ≤ 0.001, **P ≤ 0.01, and *P ≤ 0.05. For oral glucose tolerance tests, two-way ANOVA was applied to raw data to evaluate multiple comparisons between all groups and time points. iAUC was calculated using the baseline glucose value as the zero *Y* parameter; percentage reduction was calculated manually, and *t* tests were applied to verify significance between treated groups versus control. The outliers in the DIO rat model study were identified and removed using the Grubbs method (α = 0.2). For fat adiposity measurements, data were analyzed using one-way ANOVA, and the differences between means were analyzed using Tukey’s multiple comparisons.
