# Supplementary material for: Development of a targeted oral pharmacologic duodenal exclusion therapy for the treatment of metabolic diseases
Source: Sci Adv. 2025 May 30;11(22):eadu1326. doi: 10.1126/sciadv.adu1326 (PMC12124365; doi:10.1126/sciadv.adu1326)
Supplement: Supplementary file 1 — Supplementary Materials and Methods Figs. S1 to S5 [file sciadv.adu1326_sm.pdf]

Science Advances
AAAS

Supplementary Materials for

Development of a targeted oral pharmacologic duodenal exclusion therapy for the treatment of metabolic diseases

Taylor L. Carlson *et al.*

Corresponding author: Ashish Nimgaonkar, animgao1@jh.edu

Sci. Adv. **11**, eadu1326 (2025)
DOI: 10.1126/sciadv.adu1326

This PDF file includes:

Supplementary Materials and Methods
Figs. S1 to S5

Supplementary Materials and Methods

Starting materials for synthesis

Poly(allylamine hydrochloride) was obtained from Nittobo Medical, Japan (PAAn-HCl, Cat# PAA-HCl-3L, 50.3% solution in water) and used as received. The material was qualified by 1H-NMR, TGA, and size exclusion chromatography (SEC-MALLS).

1-(3-Dimethylaminopropyl)-3-ethylcarbodiimide hydrochloride (EDC-HCl, Cat# 00050, 99.8%), 1-Hydroxybenzotriazole hydrate (HOBt, Cat# 24755, 99.8% (odb), 21.3% water), and 4-Carboxyphenylboronic acid (CPBA, Cat# 28086, 99.7%) were obtained from Chem-Impex International, Wood Dale, IL. 3-Fluoro-4-Carboxyphenylboronic acid was obtained from AOB Chem, Zhuhai, China (FCPBA, Cat# 10055, 97%). All materials were used as received and qualified by 1H-NMR, FT-IR, and/or melting point.

Synthesis of PAAn modified with 4-Carboxyphenylboronic acid

PAAn-HCl 50.3% solution (5.964 g, 32 mmol of amine equivalents) was placed in a 250 ml beaker with a magnetic stir bar and deionized water (90 ml). The resulting clear solution was magnetically stirred, and a pH electrode was introduced. The pH was adjusted to 8.0 by dropwise addition of a 1N NaOH solution while stirring. 4-Carboxyphenylboronic acid (0.907 g, 5 mmol) was added to the reaction mixture and the resulting suspension was stirred. After 20 minutes of stirring, the pH had dropped to 6.8. Additional NaOH solution was added in portions with stirring to cause the complete dissolution of the suspension. The pH of the resulting clear solution was 7.5. Solid HOBt-hydrate powder (0.046 g, 0.25 mmol) was then added to the clear reaction solution and dissolved after 20 minutes of stirring. Hydrochloric acid (1N) was added to the reaction mixture to lower the pH to 5.4. EDC-HCl (1.152 g, 6 mmol) dissolved in 10 ml of deionized water was then slowly pipetted into the reaction mixture. The clear reaction mixture had a final volume of 120 ml and a pH of 5.6. This reaction mixture was stirred for 18 hours. The reaction mixture was pH adjusted to 2.5 with 1N HCl and then subjected to dialysis against a 2.5% NaCl solution using tangential flow filtration on a Pall Minimate™ TFF system. After removal of 5-diafiltration volumes and desalting until the filtrate had a conductivity of < 200 μS/cm, the retentate solution was collected in a lyophilization jar. The solution was frozen in an IPA/dry-ice slurry and lyophilized until dry.

Synthesis of PAAn modified with 3-Fluoro-4-Carboxyphenylboronic acid

PAAn-HCl 50.3% solution (5.964 g, 32 mmol of amine equivalents) was placed in a 250 ml beaker with a magnetic stir bar and deionized water (90 ml). The resulting clear solution was magnetically stirred, and a pH electrode introduced. The pH was adjusted to 8.0 by dropwise addition of a 1N NaOH solution while stirring. 3-Fluoro-4-carboxyphenylboronic acid (1.034 g, 5 mmol) was added to the reaction mixture and the resulting suspension was stirred. After 20 minutes of stirring, the pH had dropped to 6.8. Additional NaOH solution was added in portions with stirring to cause the complete dissolution of the suspension. The pH of the resulting clear solution was 7.5. Solid HOBt-hydrate powder (0.046 g, 0.25 mmol) was then added to the clear reaction solution and dissolved after 20 minutes of stirring. Hydrochloric acid (1N) was added to the reaction mixture to lower the pH to 5.4. EDC-HCl (1.152 g, 6 mmol) dissolved in 10 ml of deionized water was then slowly pipetted into the reaction mixture. The clear reaction mixture had a final volume of 120 ml and a pH of 5.6. This reaction mixture was stirred for 18 hours. The reaction mixture was pH adjusted to 2.5 with 1N HCl and then subjected to dialysis against a 2.5% NaCl solution using tangential flow filtration on a Pall Minimate™ TFF system. After

removal of 5-diafiltration volumes and desalting until the filtrate had a conductivity of < 200 µS/cm, the retentate solution was collected in a lyophilization jar. The solution was frozen in an IPA/dry-ice slurry and lyophilized until dry.

Synthesis of PAAn modified with 14C-3-Fluoro-4-Carboxyphenylboronic acid
Polymer was radiolabeled using C14 iodomethane to alkylate primary amine moieties on the allylamine backbone producing stable C14 methyl. Dose formulation was assessed to ensure radiochemical purity and stability. 14C-GLY-200 was provided by GMP-certified contract manufacturer, Moravek (Brea, CA).

Analytical characterization of polymers
Polymers were evaluated for appearance, pH (0.5 wt% in saline, Mettler Toledo pH meter), conductivity (0.5 wt% in deionized water, Mettler Toledo Seven2Go conductivity meter), viscosity (5-18 wt% in saline, Brookfield DV3T viscometer), structure conformation (1H-NMR spectrum, JEOL ECZ400, D2O/DCl), % substitution (1H-Nuclear magnetic resonance, JEOL ECZ400), molecular weight (Batch Malls), and sodium, chloride and boron content (ICP-MS, Boston Scientific, Marlborough, MA).

Collection of Porcine Mucus
Porcine small intestine was obtained from a local abattoir within 2 hours of tissue collection. The intestine was rinsed with cold water to remove food matter and drained. Mucus was collected by opening the intestine longitudinally and gently scrapping the mucosa with a spatula. Mucus was used fresh or stored at -20 °C until use.

Particle Pegylation and Characterization
FluoSpheres™ (200nm, Thermofisher, Cat# F8816) were PEG-ylated using EDC chemistry. Briefly, FluoSpheres™ were incubated with amine polyethylene glycol (MPEG-NH2, MW: 2000 Da, Laysan Bio Inc) in a 50 mM 2-(N-morpholino)ethanesulfonic acid (MES) pH 6.0 buffer for 15 min. EDC was added and the solution was adjusted to pH 6.5 then incubated for 2 hours. The solution was quenched with glycine and dialyzed overnight. The zeta potential of particles (~0.02% solids in 10 mM NaCl) was determined by Particle Technology Labs (Downers Grove, IL) with a Zetasizer Nano ZS (Malvern Panalytical).

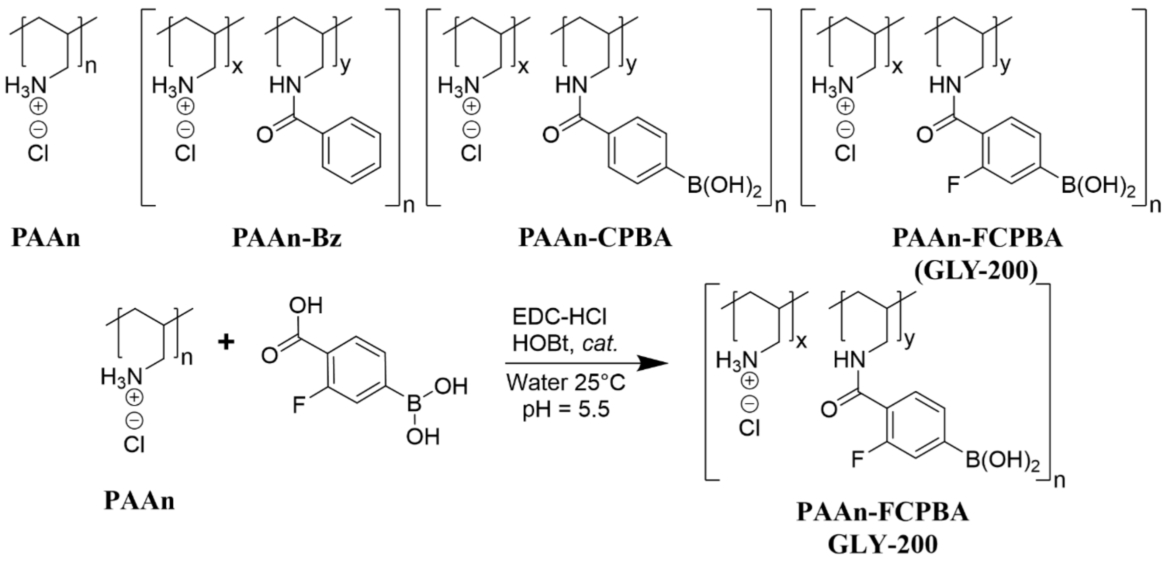

Fig. S1. Chemical structure and synthesis for PAAn and derivatives
Poly(allylamine) (PAAn) and key amide derivatives described in this study.

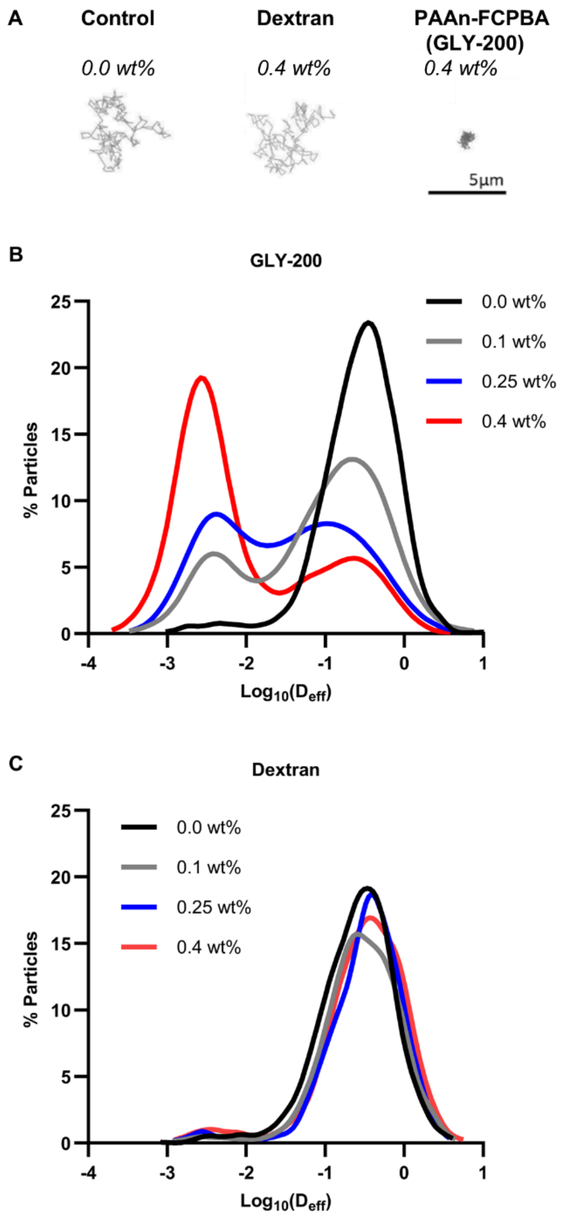

Fig. S2. Impact of polymer on mucus bulk transport properties
(A) 20-second representative particle trajectory and **(B-C)** Distribution of particle diffusivities in mucus treated with 0.0, 0.1, 0.25, and 0.4 wt% polymer.

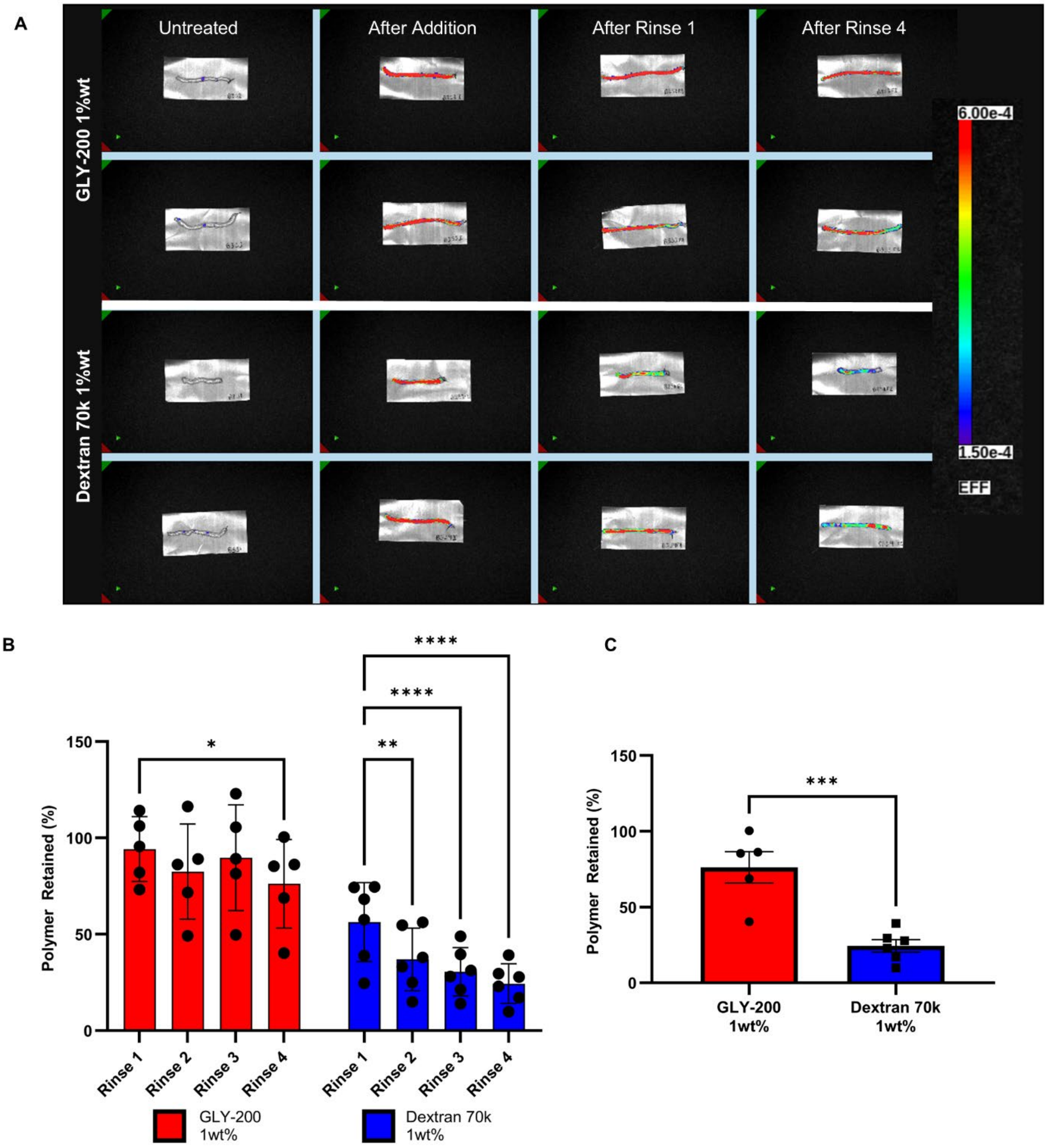

Fig. S3. Robust complexation of GLY-200 and mucin on rat tissue

A) Polymer visualized with IVIS in rat tissue (~3.5cm intestinal sections) prior to, after polymer addition, and after washing, with B) polymer retention quantified after each rinse step and C) after four rinse challenges. (n≥5) Mean ± SEM, ****p≤0.0001, ***p≤0.001, **p≤0.01, *p≤0.05.

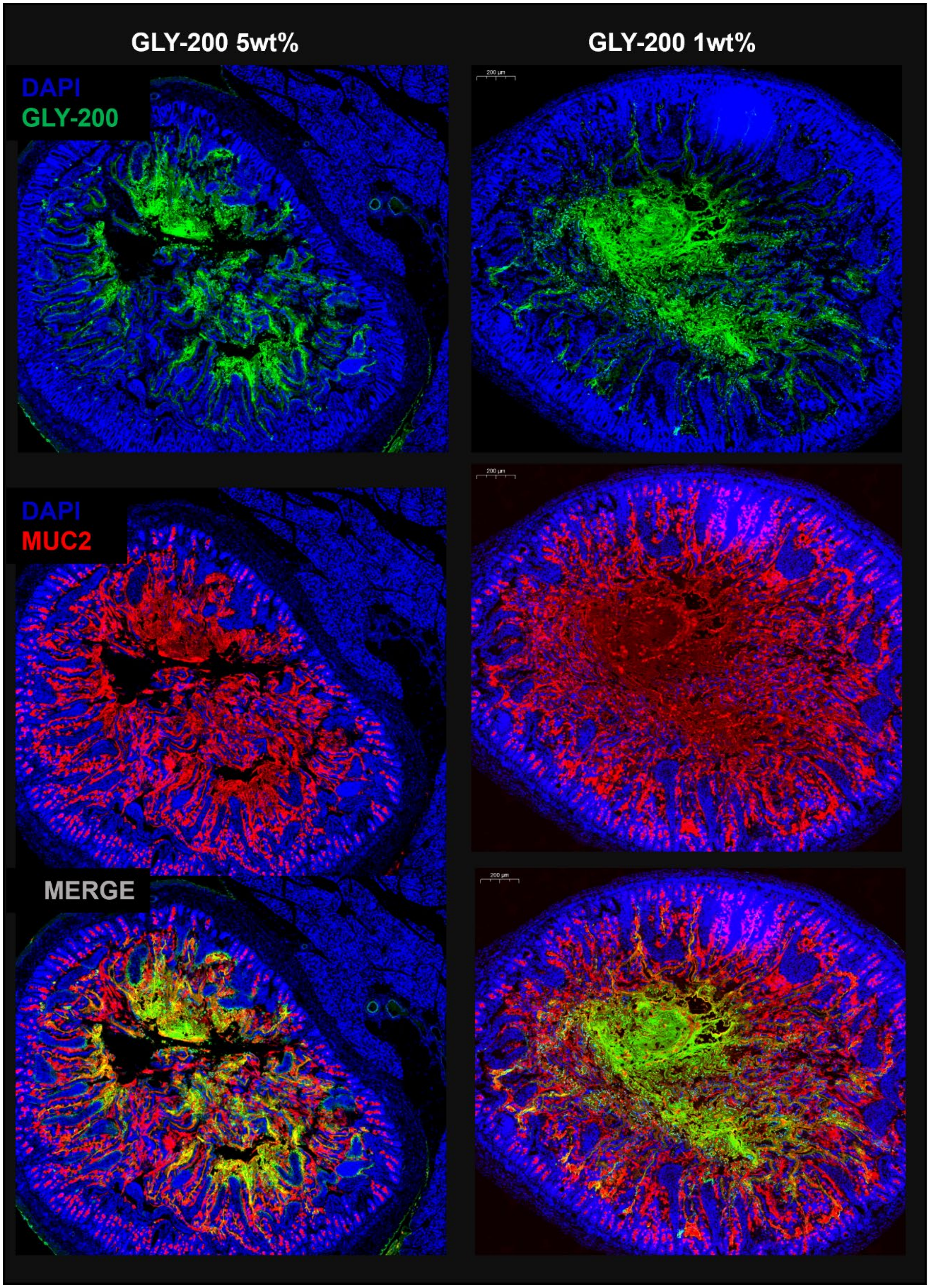

Fig. S4. Robust Polymer Signal in Rat Intestinal Tissue Following Rinse Challenge
Intestinal immunohistology cross sections visualize distribution of mucin and polymer after four rinse challenges. Blue: DAPI, Red: anti-MUC2, Green: GLY-200-FITC. Magnification 4.7x.

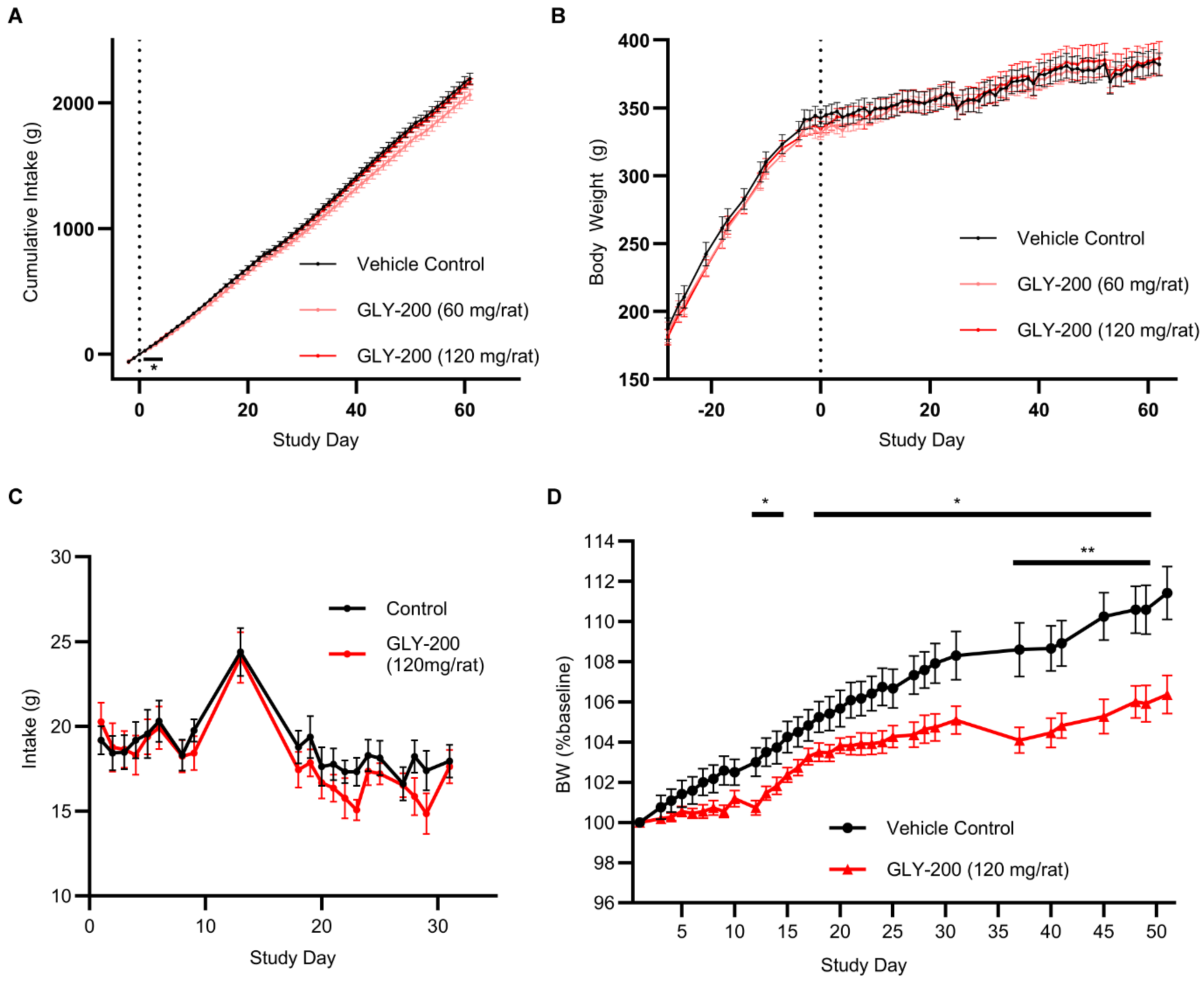

Fig. S5. Food intake and body weight following once-daily dosing of GLY-200 in ZDF and DIO rat models for 8 weeks. **(A)** Food intake and **(B)** body weight in ZDF rodents receiving chronic GLY-200 treatment (60 and 120 mg/rat/day, N=14/group), **(C)** food intake and **(D)** body weight in DIO rodents following daily dosing of GLY-200 (120mg/rat, N=11-12 / group). Mean ± SEM, **p≤0.01, *p≤0.05.
